# Evaluation of the neuroprotective, anticonvulsant, and cognition-improvement effects of apigenin in temporal lobe epilepsy: Involvement of the mitochondrial apoptotic pathway

**DOI:** 10.22038/ijbms.2019.33892.8064

**Published:** 2019-07

**Authors:** Paria Hashemi, Javad Fahanik Babaei, Somaye Vazifekhah, Farnaz Nikbakht

**Affiliations:** 1 Department of Physiology, School of Medicine and Cellular and Molecular Research Centre, Iran University of Medical Sciences, Tehran, Iran; 2 Physiology Research Centre, Iran University of Medical Sciences, Tehran, Iran; 3 Cellular and Molecular Research Center, School of Medicine, Iran University of Medical Sciences, Tehran, Iran

**Keywords:** Apigenin, Anticonvulsant, Cognition, Cytochrome c, Neuroprotection, Temporal lobe epilepsy

## Abstract

**Objective(s):**

Cognitive deficit is a common problem in epilepsy. A major concern emergent from the use of antiepileptic drugs includes their side effects on learning and memory. Herbal medicine is considered a complementary and alternative therapy in epilepsy. Apigenin is a safe flavone with antioxidant properties. However, there is little information about the beneficial effect of apigenin on cognition in epilepsy.

**Materials and Methods:**

For evaluating the anticonvulsant effect of apigenin in the kainite temporal epilepsy model, apigenin was orally administered at 50 mg/kg for six days. Reference and working memory were examined via the Morris water maze and Y-maze task spontaneously.

**Results:**

Results showed that apigenin had significant anticonvulsant activity (*P*<0.01) and restored the memory-deficit induced by kainic acid (*P*<0.05). Furthermore, apigenin significantly increased the number of living neurons in the hilus *(P*<0.001). Immunohistochemical analysis showed that apigenin reduced the release of cytochrome c (*P*<0.01), suggesting an inhibitory role in the intrinsic apoptotic pathway.

**Conclusion:**

These results suggest that apigenin restores memory impairment via anticonvulsant and neuroprotective activity.

## Introduction

Epilepsy is one of the most common and chronic neurological diseases. It manifests itself by recurring, unprovoked seizures that have no clear prognosis (1). Temporal lobe epilepsy (TLE), a type of partial or focal epilepsy, is recognized as the most frequent form of acquired epilepsy in adults (2). Partial evidence indicates that memory impairment is one of the main complaints of patients with TLE; it is caused by hippocampal sclerosis (3). Hippocampal sclerosis is characterized by the loss of neurons, specifically in the CA1/CA3 and the hilus areas of the hippocampus (4). Some studies have suggested that there is a clear interaction between memory loss and neurodegeneration in the temporal cortex, amygdala, and hippocampus (5, 6). Apparently, prolonged seizures lead to progressive hippocampal neuronal death, which is accompanied by memory dysfunction (7). 

Mitochondrial dysfunction after prolonged seizure is the other cause of neuronal cell death. One of the mitochondrial dysfunction manifestations during epilepsy is opening of the mitochondrial permeability transition pore and release of cytochrome c into the cytosol. Cytochrome c is the key protein that can trigger mitochondrial cell death (8).

Apart from seizures per se and vast hippocampal neurodegeneration, almost all available antiepileptic drugs (AEDs) worsen memory impairment (9, 10). Recent studies have shown that AEDs have not only been unsuccessful in controlling and reducing epileptic seizures in drug-resistant patients but have also led to undesirable side effects (9). Therefore, there is an urgent need to replace these with other alternatives. Recently, scholars have focused on medical herbs, especially flavonoids, on neurodegenerative disorders, including epilepsy (11). Apigenin, a natural flavone, is abundant in many fruits and vegetables, with chamomile and celery being the highest sources (12). Apigenin has received particular interest because of its anxiolytic, sedative, neuroprotective, anticonvulsive, and antidepressant properties (13). Apigenin can also ameliorate memory dysfunction linked to Alzheimer’s disease (14). The anti-cancer properties of apigenin are due to the induction of apoptosis through different apoptotic pathways (15-17). However, in pathological conditions, apigenin acts as an anti-apoptotic agent (18, 19). There are no data suggesting the protective role of apigenin in cognition improvement through amelioration of mitochondria dysfunction in TLE. Therefore, this study was designed to investigate the protective role of apigenin in cognition and memory impairments induced by kainic acid with emphasis on the possible involvement of mitochondrial cell death pathway. Additionally, we examined the anticonvulsant effect of apigenin in the rat model of TLE.

## Materials and Methods

***Animals***

Male Wistar rats (200–250 g) were obtained (n=44) from the Experimental Studies Centre, Iran University of Medical Sciences, Tehran, Iran. The animals were kept under the following standard conditions: 12 hr light/dark cycle (lights on from 7:00 AM to 7:00 PM), environmental temperature 22±2 °C, and humidity 40–50% with free availability of food and water throughout the study. All experimental protocols and procedures were compiled according to Guidelines for the Care and Use of Laboratory Animals as stipulated by the National Institute of Health (NIH). In addition, this study was approved by the Ethics and Research Committee of Iran University of Medical Sciences (Tehran, Iran) in 2016.

***Experimental procedure***

**Figure 1:**
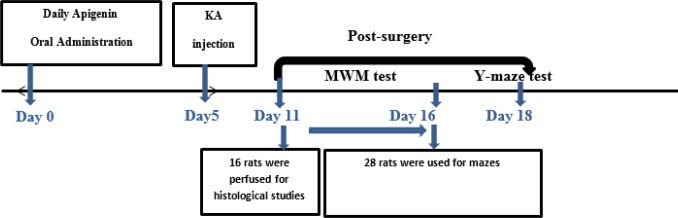
Experimental design of the study. For induction of TLE, 0.5 μg kainic acid was injected unilaterally into the left lateral ventricle. Apigenin was administrated orally at a dose of 50 mg/kg/day starting 5 days before surgery and continued 1-day post-surgery

The rats were randomly divided into 4 groups as follows: control-vehicle group, apigenin-treated sham group, kainic acid group, and apigenin-treated kainic acid group. For induction of TLE in rats, they were first anesthetized by the intraperitoneal (IP) injection of ketamine (100 mg/kg) and xylazine (20 mg/kg). Animals were then positioned and fixed on a stereotaxic apparatus (Stoelting, USA). After a midline sagittal incision in the scalp, the left lateral ventricle was targeted at the anteroposterior position, in accordance to the stereotaxic atlas, as follows: 1 mm; lateral; –1.5 mm from bregma, and 3.5 mm ventral from the dura. An amount of 1.8 μl of sterile normal saline solution containing 0.5 μg of kainic acid was microinjected unilaterally into the left lateral ventricle. After 5 min, the needle was removed to minimize the drug withdrawn. Apigenin was dissolved in distilled water, containing 5% sodium-carboxyl methylcellulose (CMC-Na) and was administered orally at a dose of 50 mg/kg/day once a day five days before surgery till one day afterward (Figure 1).

***Behavioral testing***

*Anticonvulsant activity of apigenin against kainite-induced seizures*

In kainite-induced convulsion, the intensity of the seizures, the onset of seizures, the onset of tonic-clonic general seizure, and the duration of tonic-clonic phase were video monitored and recorded for 24 hr.

Seizure intensity was measured according to a modified Racine’s scale values:

Stage 0: immobility; stage 1: rigid posture, eye blinking, and /or mild facial clonus; stage 2: head bobbing and multiple facial clonus; stage 3: myoclonic jerks in the forelimbs; stage 4: clonic convulsions in the forelimbs with rearing; and stage 5: generalized tonic-clonic convulsions.

*Training in the spatial version of the Morris water maze task*

Morris water maze (MWM) is one of the most commonly accepted behavioral tests for assessing the spatial learning and memory capabilities of rodents. In the present study, MWM was conducted five days after induction of TLE in rats. MWM was a circular black tank (100 cm in diameter and 75 cm in height). It was placed in a dim room with visual cues. The tank was filled to a depth of 40 cm with water (21±1 °C). A hidden black platform (10 cm in diameter) was constantly located 1.5 cm beneath the water surface in one of the pool quadrants. The task consisted of 2 stages: the acquisition phase for spatial learning assessment, and the probe test and retention retrieval of memory. The acquisition phase was performed during five subsequent days, in which the rats were given four trials per session per day with the platform in a constant place. In each session and between each trial, an interval of 20 sec was observed. If the platform was not found within 60 sec, the rat was forced into the platform and placed on it for an additional 20 sec. During each trial session, the total time (latency time) and movement to find the hidden platform was recorded using a video camera-based EthoVision System (Noldus, Netherlands). Twenty-four hours after the acquisition phase, the probe trial session was performed in which the platform was removed from the pool and the rat was allowed to swim for 60 sec to search for it. The latency of first entry to the target quadrant, time spent in the target quadrant, and the total number of crossings into the target quadrant were recorded. 

*Y-maze spontaneous alternation performance*

This test was conducted 2 days after the MWM task. To evaluate spatial working memory, we recorded spontaneous alternation behavior during a single-session in a Y-maze on the 13th day of the experiment. Testing occurred in a Y-shaped maze which consisted of a gray-painted Plexiglas with three horizontal arms (40 cm long, 30 cm high, and 15 cm wide). The rat, without any prior experience of the maze, was put at the start arm and then allowed to move freely inside the maze for a 10-min session. The arm entries were counted and recorded visually. Alternation rate was defined as entries into all three arms on consecutive occasions using the following formula:

Alteration rate (%)=Number of alterations / (Number of total arm entries–2) × 100

*Histological processing*

Five days after the KA injection, four rats in each group were anesthetized with ketamine (100 mg/kg) and xylazine (20 mg/kg): they were then perfused via the left cardiac ventricle with 4% paraformaldehyde in 0.1 M phosphate-buffered saline (pH 7.4). The brains were removed and post-fixed overnight at 4 °C. Twenty four microtome serial sections of each brain from the hippocampus area (5 μm) were used for Nissl, Fluoro-Jade B, and cytochrome c immunoreactivity. Eight sections with three sections’ interval of each brain were used for each histological process. For Nissl staining, the slides were stained with 0.1% Cresyl violet for 1 min after dehydration in graded ethanol solutions: they were then rehydrated and coverslipped. The number of Nissl-stained granule cells in CA3 and the hilar areas of the hippocampus were counted using a 20X objective.

The second eight sections were used for Fluoro-Jade B staining according to the Schmued (2000) protocol (20). In brief, the slides were immersed in xylene: 100% ethanol for 3 min, followed by 2 min in 70% ethanol. Then, the sections were placed in a solution containing 1% sodium hydroxide in 80% alcohol. Subsequently, they were rinsed in distilled water for 2 min before being transferred to a solution of permanganate for 10 min. Following rinsing in distilled water, the sections were immersed into a 0.004 Fluoro-Jade B working solution for about 30 min in darkness. The sections were then air dried in the dark for 20 min. Finally, the dry slides were cleared in xylene and coverslipped with DPX, a non-aqueous mounting medium. For observation of neuronal degeneration, results were assessed using the FITC filter at 40X magnification.

Eight sections were used for cytochrome C immunoreactivity. For this purpose, endogenous peroxidase activity was blocked in a dark room for 20 min in 3% H2O2/methanol solution after dehydration in graded ethanol solutions. After washing with PBS, they were heated indirectly for 30 min in a bain marie (70 °C) and then treated with blocking serum consisting of 3% goat serum and 0.3% Triton X-100 for 20 min. The sections were incubated overnight with 4 °C primary antibodies at 1:100 dilutions (anti-cytochrome c rabbit polyclonal antibody). Sections were incubated for 60 min at room temperature with goat anti-rabbit IgG HRP conjugated as a secondary antibody. Chromogenic 3,3′-diaminobenzidine (DAB) solution was used for detecting cells, while Mayer’s hematoxylin (Thermo Scientific Inc., Waltham, MA, USA) was used for counterstaining.

***Statistical analysis***

The results were expressed as mean±SEM. Differences were considered statistically significant at *P*<0.05. Statistical analyses were performed using SPSS v.16.0 for windows. The data related to MWM were analyzed by two way ANOVAs. MWM probe test data and other studies were analyzed using one-way ANOVA followed with subsequent Tukey’s *post hoc* test to analyze the difference. 

## Results

***Anticonvulsant activity of apigenin***

**Figure 2:**
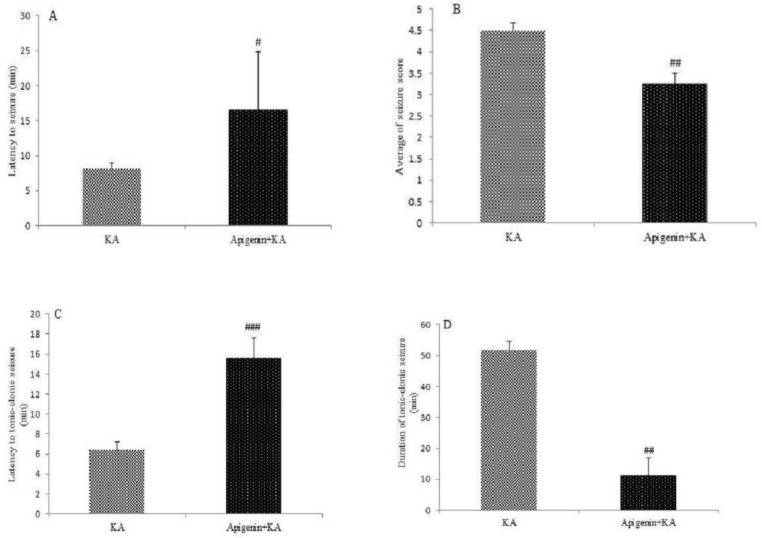
Anticonvulsant effect of apigenin (50 mg/kg/day). Following apigenin treatment, decrease in the onset of seizure (*P*<0.05; A), average seizure intensity (*P*<0.01; B), latency to the tonic-clonic stage (*P*<0.001; C), and duration time in tonic-clonic stage (*P*<0.01; D) were recorded. The data represented as the mean±SEM, statistically significant differences compared with KA group

The results show that apigenin has an anticonvulsant effect on KA-induced seizure activity. Apigenin significantly delayed the latent period to start seizure as compared with the KA group (*P*<0.05) (Figure 2A). Administration of apigenin significantly decreased the severity of the seizure recorded by the Racine scale (*P*<0.01, Figure 2B) in comparison with the KA group. Also, apigenin decreased the threshold and duration of tonic-clonic stages (*P*<0.001 and *P*<0.01, respectively).

***Effect of oral administration of apigenin on learning and memory deficits induced by KA in the Morris water maze task***

*Effect of apigenin on acquisition phase of MWM*

**Figure 3:**
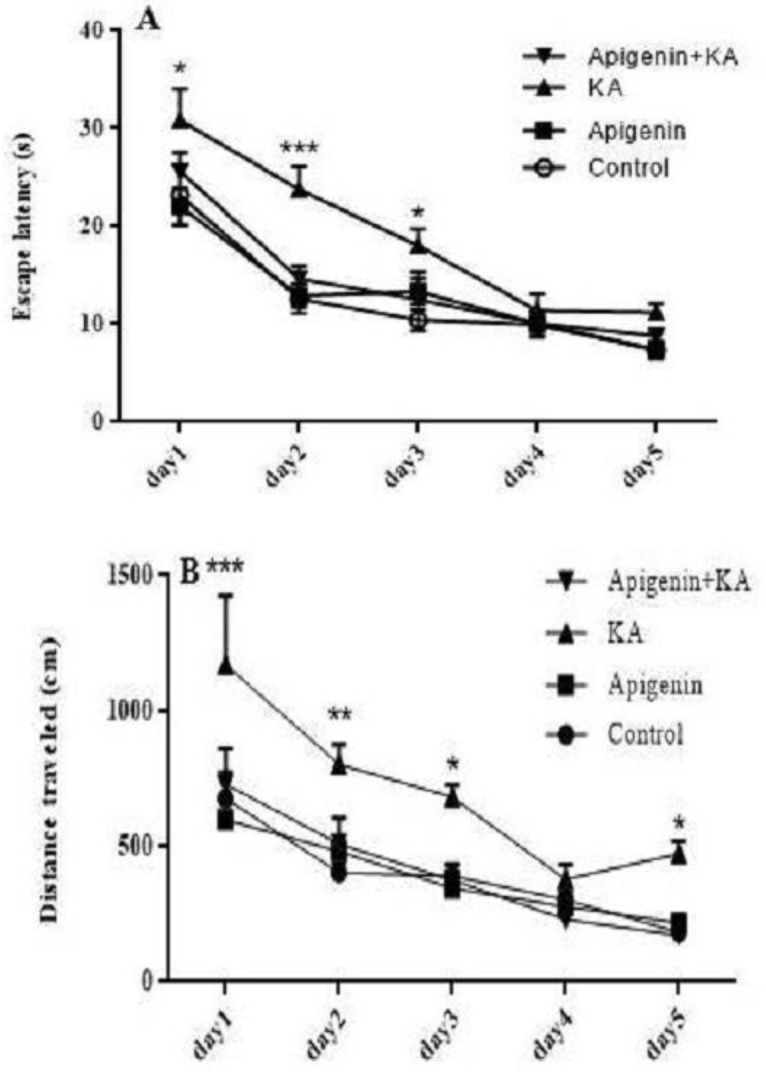
Effect of apigenin (50 mg/kg/day) on the acquisition phase of the Morris water maze (MWM) test. (A) Mean escape latency to reach the platform during the acquisition phase, (B) total distance traveled to reach the platform. Apigenin treatment significantly ameliorated learning-deficit induced by kainite All data are expressed as mean±SEM

Figure 3 shows the results of learning in the acquisition phase of MWM. The latency to reach the platform during the learning phase was analyzed by two-way ANOVA. It revealed that there was a significant influence of the factor days [F (4, 140): 56.05, *P*<0.001] and groups [F (3, 140): 13.76, *P*<0.001], but not for the interaction of the day group [F (12, 140): 1.19, *P*=0.29]. When this interaction was analyzed with one-way ANOVA, it was evident that KA rats used significantly longer latencies to reach the platform as compared with the control on days 1, 2, and 3 (*P*<0.05; *P*<0.001; and *P*<0.05 respectively). Pre-treatment of the KA group with apigenin significantly decreased escape latency as compared with the KA group only on Day 2 (*P*=0.01). There was no significant difference between groups on days 4 and 5 (Figure 3A).

Regarding the total distance traveled, the same statistical analysis revealed significant effect on both factors: days [F (4,140): 30.04, *P*<0.001] and groups [F (3, 140): 19.41, *P*<0.001]. However, the interaction between days and groups failed to reach a statistical significance [F (2, 140): 1.005,* P*=0.44]. One-way ANOVA further revealed an increase in the total distance traveled in the KA group as compared with the control group on day 1 (*P*<0.001), day 2 (*P*=0.01), and days 3 and 5 (*P*=0.05). Pre-treatment with apigenin significantly decreased distance traveled as compared with the KA group on these days [1 (*P*<0.001), 2 (*P*=0.01), and 3 and 5 (*P*=0.05)]. Apigenin treatment in the sham group did not show any significant enhancement on learning as compared with the control rats, implying that apigenin alone cannot improve memory in normal rats (Figure 3B).

***Effect of apigenin on retrieval phase of MWM***

**Figure 4:**
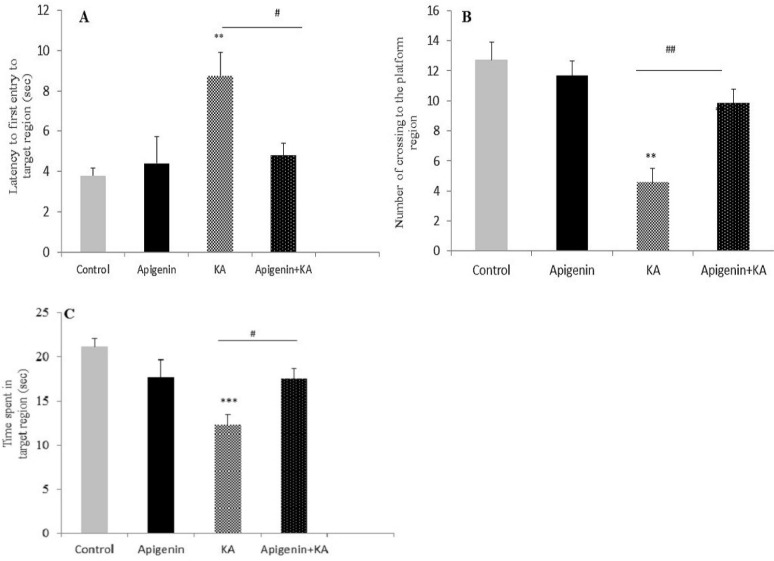
Effect of apigenin (50 mg/kg/day) on the retrieval phase of the Morris water maze (MWM) test. The statistical analysis of data from first entry to the target quadrant (A), the number of crossings into the target region (B), and the total time spent in the target region (c) revealed significant impairment in memory retrieval after status epilepticus. However, apigenin restored memory in apigenin-treated groups

Probe trial test was conducted to assess memory retrieval 24 hr after the last training session. Three parameters of first entry to the target quadrant, the number of crossings into the target quadrant, and the total time spent in the target region were analyzed using one-way ANOVA. These results reveal a significant effect caused by treatment on latency to the first entry to the target quadrant [F (3, 27): 6.30, *P*=0.003]. *Post hoc* test confirmed that the KA group had higher latency upon first entry to the target quadrant compared with control and apigenin-treated sham groups (*P*=0.009, *P*=0.003 respectively; Figure 4A). Apigenin treatment significantly reduced latency to the first entry as compared with the KA group *(P*<0.05). The number of crossings into the target region in different groups revealed a significant difference between groups [F (3, 27): 15.99; *P*<0.001, Figure 4B). *Post hoc* analysis confirmed that KA rats showed a lower number of crossings into the target region (where the platform had been located before) as compared with the control and apigenin-treated groups (*P*<0.01). One-way ANOVA also showed that there was a significant difference between all groups for the total time spent in the target quadrant [F (3, 27): 7.69; *P*=0.001, Figure 4C]. *Post hoc* analyses indicated less time spent in the KA group as compared to the control group (*P*<0.001): this was ameliorated by apigenin in the treated KA group (*P*<0.05). As there was no difference between the apigenin treated group and the control group, we conclude that the reference memory impairment induced by kainite was restored by apigenin.

***Effect of oral administration of apigenin on spatial working memory in KA-induced rat model of TLE***

**Figure 5:**
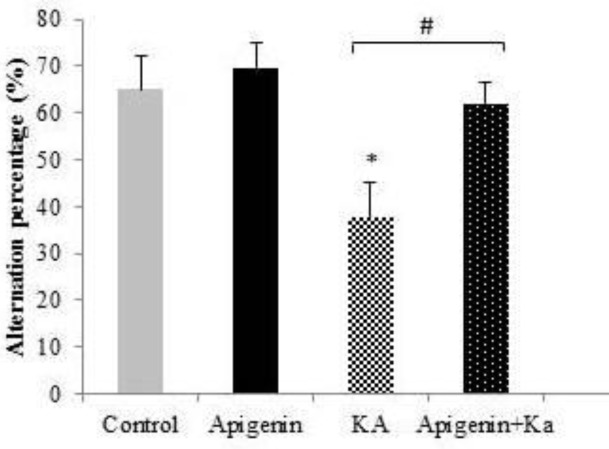
Effect of apigenin (50 mg/kg/day) on spatial working memory shown by the percentage of alternative behavior in the Y-maze task. Apigenin treated rats restored the working memory deficit induced by KA. *P<0.05 (vs control); #*P*<0.05 (vs KA)

Significant differences were detected amongst the experimental groups regarding the Y-maze task using the one-way ANOVA test. As shown in Figure 5, the mean percent spontaneous alternation behavior for the control-vehicle, apigenin-treated sham, kainic acid, and apigenin-treated + kainic acid was 65±6.99, 69.47±5.53, 37.53±7.58, and 61.99±4.4, respectively. Thus, a significant decrease in the percent alternation behavior was observed in the KA group as compared with the control and apigenin-treated sham groups (*P*<0.05). Treatment of the KA group with apigenin significantly increased the percentage alternation behavior (*P*<0.05): this indicates that apigenin restores the working memory deficit induced by kainite. 

***Effect of oral administration of apigenin on neuronal loss and neurodegeneration in KA-induced rat model of TLE***

**Figure 6:**
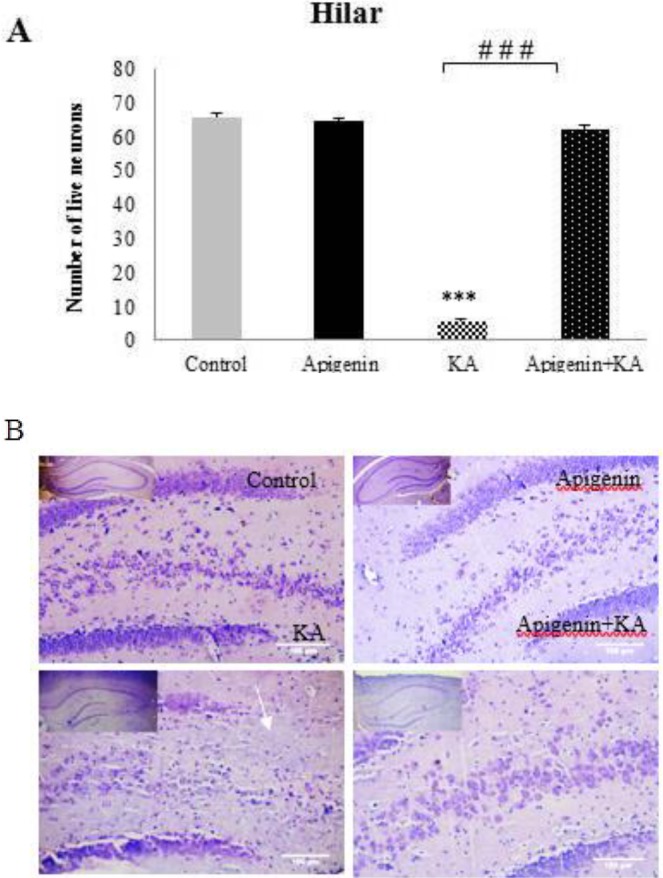
Effect of apigenin (50 mg/kg/day) on neuronal loss in the hippocampus

**Figure 7:**
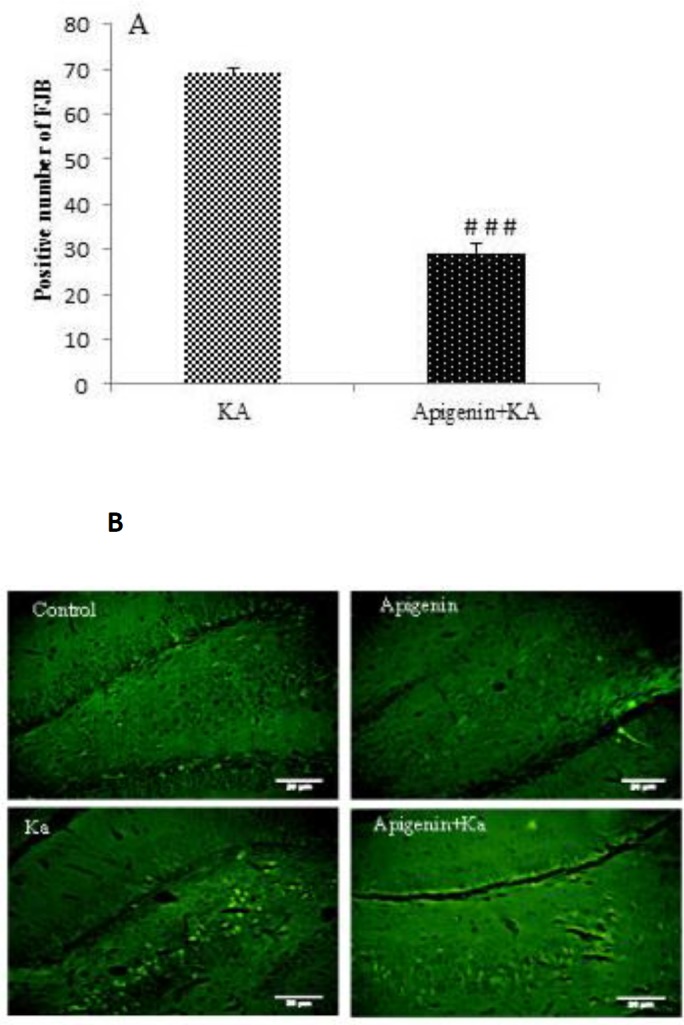
Effect of apigenin on neurodegeneration in the hippocampus. (A) Quantitative analysis showed a significant decrease in Fluoro-Jade B positive cells in apigenin-treated compared to ka group .### P<0.001 by one-way ANOVA. (B) Representative sections of Fluoro-Jade B staining in hilus region of hippocampus of ka and apigenin+ka groups. Abundant Fluoro-Jade B positive neurons were seen in ka group, but to a lesser degree in apigenin-treated rats. Scale Bar = 100 μm

In the present study, the number of live neurons using Nissl staining and neurodegeneration using Fluoro-Jabe B in the hilar area of the hippocampus of all experimental groups was counted and analyzed (Figures 6 & 7). Data showed that intracerebroventricular microinjection of KA leads to a significant neuronal loss, *P*<0.001. As shown in Figure 6, in the hilar area compared with the control and apigenin-sham operated groups, apigenin treatment of the KA group at a dose of 50 mg/kg for 5 days before induction of epilepsy significantly prevented this reduction as compared to the KA group (*P*<0.001; Figures 6 & 7).

***Effect of oral administration of apigenin on cytochrome*** ***c, mitochondrial cell death pathway in KA-induced rat model of TLE***

**Figure 8:**
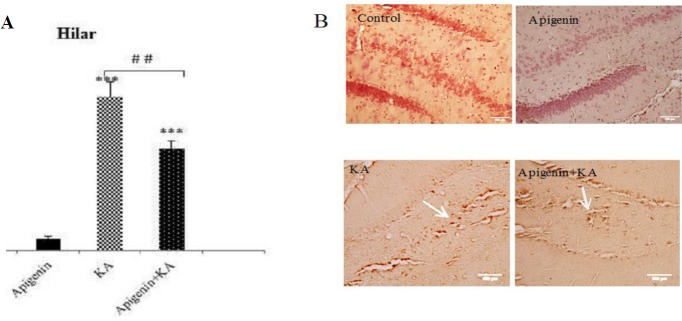
Effect of apigenin on Cytochrome C release in hilar area

We further conducted cytochrome c immunohistochemistry to evaluate whether apigenin could protect hippocampal neurons against the deleterious effect of cytochrome c and proapoptotic mitochondrial cell death pathway. In this regard, cytochrome c immunoreactivity in hilar neurons was significantly greater in KA than in the control group (*P*<0.001; Figure 8). Although apigenin treatment significantly reduced the release of cytochrome c (*P*<0.01 vs KA), there was still a significant difference between control and apigenin treated groups (*P*<0.001).

* *P*<0.05, ** *P*<0.01, *** *P*<0.001 (KA vs control)

All data are expressed as mean±SEM. ** *P*<0.01, *** *P*<0.001 (KA vs control) ; # *P*<0.05, ## *P*<0.01 (apigenin+kA vs KA)

(A) Quantification of Nissl-positive cells revealed significant neuronal damage in the hilus of the hippocampus. However, significant protection after apigenin treatment was observed.

(B) Representative images showing Nissl staining in the hilar area of the hippocampus. Scale bar = 100 μm

***P<0.001 (KA vs control); ### *P*<0.001 (apigenin+KA vs KA)

(A) Ka significantly increased the cytochrome c positive cells in the hilus of hippocampus. This effect was ameliorated after apigenin treatment

***P<0.001 (vs. control); ## *P*<0.01 (apigenin+ ka vs. ka)

(B) Immunohistochemistry of caspase-3 positive cells in the hippocampus from rat under apigenin treatments

Scale Bar = 100 μm

## Discussion

In this study, anticonvulsant cognitive improvement and neuroprotective effects of apigenin in a rat model of TLE have been investigated, and the possible involvement of the mitochondrial apoptotic pathway due to the inhibitory role of apigenin on cytochrome c release has been assessed. The improvement of learning and memory by apigenin has been shown in some neurodegenerative diseases, considering the anti-oxidative and neuroprotective properties of apigenin as the main reason for memory enhancement (14, 21). Apigenin can also stimulate neurogenesis (22): however, in our study, no memory improvement was observed in the apigenin group. To our knowledge, no study till now has revealed the effect of apigenin on memory deficit induced by epilepsy. Apart from antiepileptic drugs, recurrent seizure and hippocampal neuronal loss are the two important causes of memory impairment in epilepsy. 

Recurrent seizures have been found to be involved in cognition and memory deficits in patients with epilepsy (23). In fact, a close correlation between the degree of memory insufficiency and seizure frequency has been shown (24). In the present study, apigenin showed anti-seizure activity which may, to some extent, be responsible for improvement in the memory performance following apigenin pretreatment. This is consistent with the findings of Han *et al*., who showed that apigenin has anticonvulsant activity in kainate-induced seizure due to its anti-oxidative properties (25). On the other hand, apigenin can interact with gamma-aminobutyric acid type A (GABA_A_) receptors, suggesting an enhancement of the GABAergic system (26). Apigenin can also inhibit glutamate release in rat hippocampus (27). Therefore, the other probable mechanism to explain the anticonvulsant activity of apigenin is through exerting a balance between the inhibitory and excitatory neurotransmitters in the hippocampus. 

It has been documented that seizures result in excessive neural loss and hyperactivity of the apoptotic pathways (28), especially in the hippocampus: this leads to memory deficit in TLE patients. Neural cell loss in the CA3/CA1 and hilar regions of the hippocampus can be directly correlated with memory loss in epilepsy (29). Both intrinsic and extrinsic apoptotic pathways initiate after seizures (28). Cytochrome c release from the mitochondria intermembrane space has been the major focus of the intrinsic pathway. In this study, hippocampal neuronal loss and cytochrome c release were found to be decreased in the apigenin-treated group: this suggests a neuroprotective role for apigenin via attenuation of the mitochondrial apoptotic pathway. In agreement with our results, data of a study has shown that apigenin can protect against the induced pluripotent stem cell model of Alzheimer’s disease by blocking caspase-3 activity (30). 

Our data not only shows the anti-apoptotic ability of apigenin but also reveals its protective role on mitochondria. 

## Conclusion

We concluded that apigenin can exert a protective effect on memory-deficit induced by kainite through anticonvulsant and anti-apoptosis activity. Overall, this study suggests that apigenin offers great potential for further investigation as a therapeutic agent in the management of memory decline in TLE.
